# Supplementary material for: Netcombin: An algorithm for constructing optimal phylogenetic network from rooted triplets
Source: PLoS One. 2020 Sep 18;15(9):e0227842. doi: 10.1371/journal.pone.0227842 (PMC7500971; doi:10.1371/journal.pone.0227842)
Supplement: S4 File — (ZIP) [file pone.0227842.s004.zip › SequenceData-Triplet/sequenceData-10-20-40-Triplet2/data-(sample 10- length 500- num seq 40- m rate 10^-6)/4 seq.pdf]

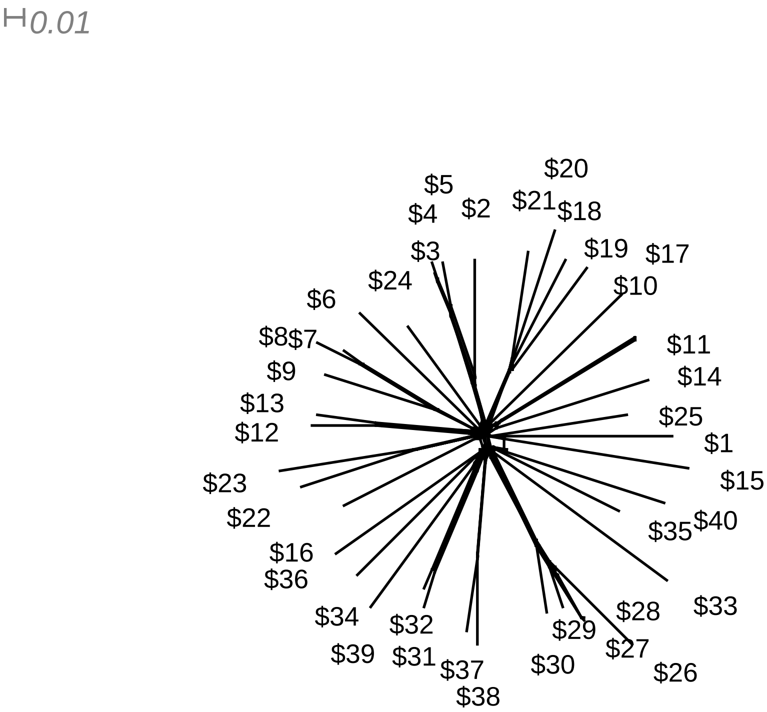

0.01
$20
$5
$21
$2
$18
$4
$19
$17
$3
$10
$24
$6
$11
$8
$7
$14
$9
$13
$25
$12
$1
$15
$23
$22
$40
$35
$16
$36
$33
$28
$34
$32
$29
$39
$31
$27
$37
$30
$26
$38
